# PROs for RARE: protocol for development of a core patient reported outcome set for individuals with genetic intellectual disability

**DOI:** 10.1186/s13023-024-03264-0

**Published:** 2024-09-27

**Authors:** Nadia Y. van Silfhout, Maud M. van Muilekom, Clara D. van Karnebeek, Lotte Haverman, Agnies M. van Eeghen

**Affiliations:** 1 Department of Child and Adolescent Psychiatry & Psychosocial Care, Emma Children’s Hospital, Amsterdam UMC location University of Amsterdam, Amsterdam, The Netherlands; 2 Department of Pediatrics, Amsterdam Gastroenterology Endocrinology Metabolism, Emma Children’s Hospital, Amsterdam UMC location University of Amsterdam, Amsterdam, The Netherlands; 3 Emma Center for Personalized Medicine, Amsterdam UMC, Amsterdam, The Netherlands; 4 Amsterdam Public Health Research Institute, Mental health and Personalized Medicine, Amsterdam, The Netherlands; 5 Amsterdam Reproduction & Development Research Institute, Child Development, Amsterdam, The Netherlands; 6 Amsterdam Public Health Research Institute, Mental health and Digital Health, Amsterdam, The Netherlands; 7 Amsterdam Public Health Research Institute, Aging & Later life and Personalized Medicine, Amsterdam, The Netherlands; 8 Advisium’s Heeren Loo, Amersfoort, The Netherlands

**Keywords:** Rare genetic neurodevelopmental disorders, Intellectual disability, Patient reported outcomes, Patient reported outcome measures

## Abstract

**Introduction:**

Rare genetic neurodevelopmental disorders and intellectual disability (ID), collectively called genetic ID (GID), can profoundly impact daily functioning and overall well-being of affected individuals. To improve our understanding of the impact of GID and advancing both care and research, measuring relevant patient reported outcomes (PROs) is crucial. Currently, various PROs are measured for GID. Given the shared comorbidities across disorders, we aim to develop a generic core PRO set for children and adults with GID.

**Methods and results:**

Developing the generic core PRO set entails the following steps: 1) providing an overview of potentially relevant PROs by scoping reviews and qualitative research; 2) integrating and conceptualizing these PROs (i.e., describing the content of the PROs in detail) into a pilot generic core PRO set; and 3) prioritizing relevant PROs by a European Delphi survey and consensus meetings.

**Conclusions:**

This protocol presents the steps for developing a generic core PRO set for children and adults with GID. The next step involves selecting suitable patient reported outcome measures (PROMs) to adequately measure these PROs: the generic core PROM set. This generic core PROM set needs validation in the GID population, and eventually implementation in care and research, facilitating the aggregation and analysis of PRO data and guaranteeing continuous integration of the patient perspective in both care and research.

**Supplementary Information:**

The online version contains supplementary material available at 10.1186/s13023-024-03264-0.

## Background

Rare genetic neurodevelopmental disorders occur in about 1% of the general population [1, 2]. By European definition, disorders are classified as ‘rare’ when affecting less than one in 2000 individuals [3, 4]. Rare genetic neurodevelopmental disorders can be associated with intellectual disability (ID) [5–7], which is defined as an IQ < 70 and affects up to 2.5% of the population [8]. With current techniques, a genetic or other biological etiology can now be identified in about half of the individuals with ID. Genetic etiology varies from point mutations in one of the more than 1500 ID-related genes identified [9–11], to chromosomal copy number variants encompassing multiple genes, short tandem repeat expansion, or other structural variation to epigenetic anomalies. Rare genetic neurodevelopmental disorders and ID, henceforth called together genetic ID (GID), often have a negative impact on daily functioning and overall well-being of affected individuals due to the wide spectrum of physical and neuropsychiatric manifestations [12, 13]. These manifestations may include pain, epilepsy, psychiatric disorders such as autism spectrum disorder, and complex behaviors such as self-injurious behavior and aggression [14–17]. Due to these complex manifestations, individuals often require lifelong support on all life domains, including intensive medical and psychological care [18].

Understanding how the manifestations of GID affect daily functioning is an important part of disease management and crucial for delivering the best possible care for individuals with GID. To gain more insight into the impact of GID on daily functioning, measuring relevant patient reported outcomes (PROs) is essential. PROs refer to aspects of a patient’s health status (e.g., pain, anxiety, or fatigue) that are directly reported by the patient themselves or a proxy (e.g,. caregiver), without interference of a clinician or someone else [19]. PROs can be multidimensional, encompassing multiple PRO constructs (e.g., perceived health), or unidimensional, including only one specific PRO construct (e.g., pain or anxiety). PROs can be measured with patient reported outcome measures (PROMs) which are standardized questionnaires completed by the patient or a proxy (e.g., caregiver) [20]. PROMs can be generic (i.e., measuring health concepts that are relevant to a broad range of conditions or the general population, such as the Pediatric Quality of Life Inventory [21]), condition-specific (i.e., measuring health concepts relevant to a specific condition, such as the tuberous sclerosis complex (TSC) PROM [22]), or individualized (i.e., measuring health concepts relevant to a specific individual, such as the Patient-Specific Complaint Questionnaire [23]) [24, 25] (see Additional file 1).

**Fig. 1:**
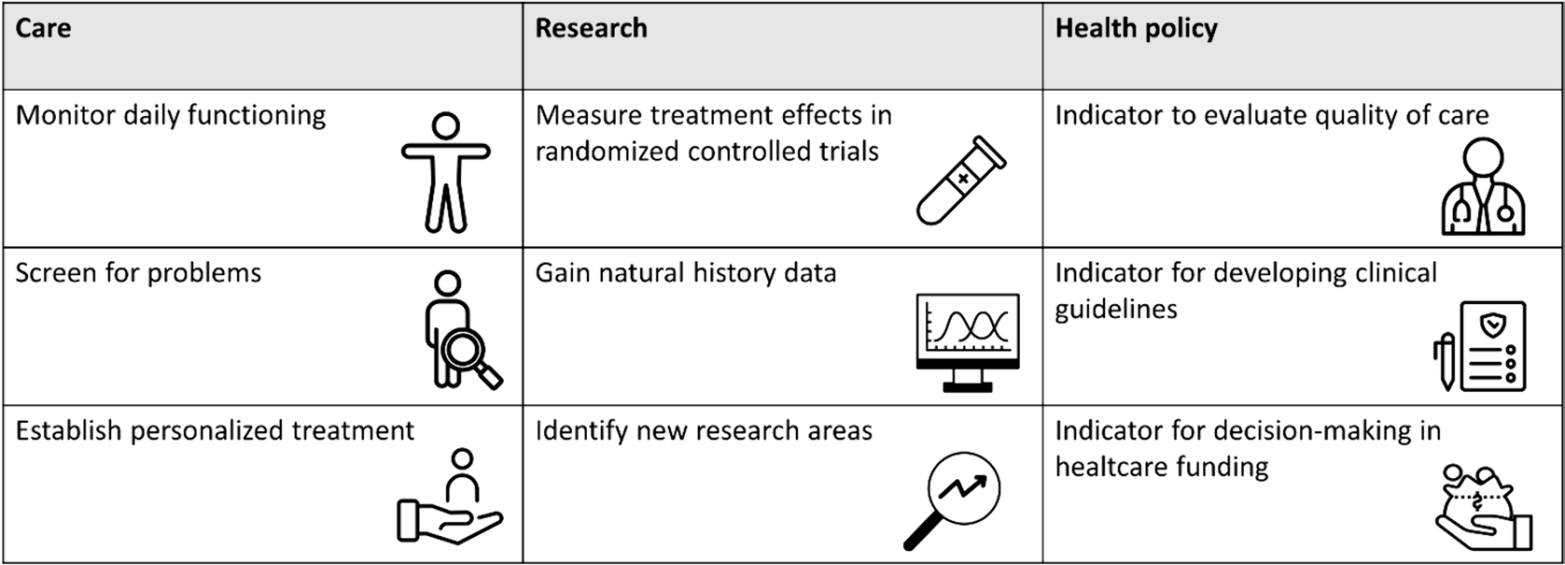
Potential benefits of utilizing PROMs in care, research, and health policy

PROMs can be used in both care and research and may serve as a valuable tool to guide health policy (Fig. 1). In care, PROMs can be used for the individual with GID to monitor overall functioning, screen for problems, and establish personalized treatment. The use of PROMs in care has already shown benefits within a wide range of fields like oncology, primary care, and psychiatry [26, 27], but is still not routinely used in care for GID [28]. In research involving individuals with GID, PROMs can be used to assess the efficacy and safety of treatments, as well as to gain more insight into the natural course of the disorder. This also helps in identifying new research areas, all from the perspective of the affected individuals or their caregivers [29, 30]. Moreover, PROMs are gaining escalating significance in patient registries for rare diseases, including GID, due to the growing recognition of the necessity to incorporate patients' perspectives to fully capture the burden of a disease and its treatment [31]. Lastly, PROMs can contribute to a more patient-centered health policy, ensuring that health policies are in harmony with the needs of patients. PROMs could, for instance, serve as an indicator to evaluate quality of the often time- and labor-intensive care delivered for individuals with GID [32]. Additionally, PRO data gathered with PROMs could inform the development of clinical guidelines and influence decisions regarding funding healthcare [24, 33].

Currently, various PROs are measured for GID, especially in research. A recent study identified more than 200 different PROMs, which were being utilized in 312 clinical trials involving individuals with GID [34]. Although this overload of PROs measured is not surprising due to the wide spectrum of manifestations of GID, it does hamper the possibility to combine and compare PRO data across different genetic or other subgroups [30]. Additionally, analyzing and interpreting PRO data can be complex due to the multidimensionality of PROs (e.g., adaptive functioning), differences in terminology used for similar PROs (e.g., participation, engagement in meaningful activity, community involvement), and unclear PROs due to lack of definition (e.g., syndrome-specific symptoms). Lastly, it is unknown whether the PROs measured are relevant to individuals with GID.

To overcome these challenges, we aim to develop a comprehensive, universally applicable core PRO set for both children and adults with GID to be used in care and research. The frequently shared comorbidities in GID can result in similar patient reported problems across disorders, enabling the development of a generic core PRO set. This corresponds to the trend seen toward standardized measurement of generic PROs across conditions in the general population, where PROs such as anxiety, depression, pain, and fatigue are often shared [35]. In this protocol, the steps for developing the generic core PRO set are described, following the Core Outcome Set-Standardized Protocol Items (COS-STAP) Statement [36] in order to align with the official steps for developing a Core Outcome Set (COS).

## Methods and results

### Stakeholders

The project team consists of a Study Management Group and a Study Advisory Board. The Study Management Group consists of researchers; one researcher (NvS) who will coordinate the day-to-day management of the project, two PROM experts and medical psychologists (MvM, LH), and two GID experts including an ID physician and a genetic metabolic pediatrician (AvE, CvK). The Study Advisory Board consists of GID experts. The Study Management Group will also collaborate with patient representatives and experts of the European Reference Network on Intellectual Disability, TeleHealth and Congenital Anomalies (ERN-ITHACA), which is a patient-centered network that is established with the goal of improving collaboration on rare neurodevelopmental disorders, both with and without biological diagnosis. Throughout this study, individuals with GID and their caregivers will be actively engaged and included where feasible and relevant. Individuals with ID without known genetic etiology will also be included within this study, since they exhibit overlapping comorbidities with GID. They will be subsumed under the term GID.

### Conceptual framework

PROs will be classified within a conceptual framework (Table 1), based on the model of Valderas and Alonso [25] (combination of the classification model by Wilson and Cleary [37] and the International Classification of Functioning, Disability and Health (ICF) [38]), and the Patient Reported Outcomes Measurement Information System (PROMIS) [39]. This model is further refined during expert meetings regarding PRO(M)s in the Netherlands [35]. A PRO domain represents an overarching PRO (e.g., physical functioning), and a PRO subdomain represents a PRO that falls under a PRO domain (e.g., mobility). Each symptom (e.g., pain) represents its own a PRO domain. The PRO conceptualization represents the description of the content of a PRO (e.g., ability to perform daily activities). Describing PROs in as much detail as possible before selecting PROMs is essential [40]. This conceptual framework should enable the classification of all PROs for GID.

**Table 1:** Conceptual framework

	**PRO domain**	**PRO subdomain**	**PRO conceptualization**
*Overarching*	Quality of life		
	Perceived health		
*Functioning*	Physical functioning		
	Social functioning/participation		
	Mental functioning		
*Symptoms*			

*PRO* patient reported outcome

PRO domain is an overarching PRO; PRO subdomain is a PRO within a PRO domain; PRO conceptualization is the detailed description of the content of a PRO

### Study design

**Fig. 2:**
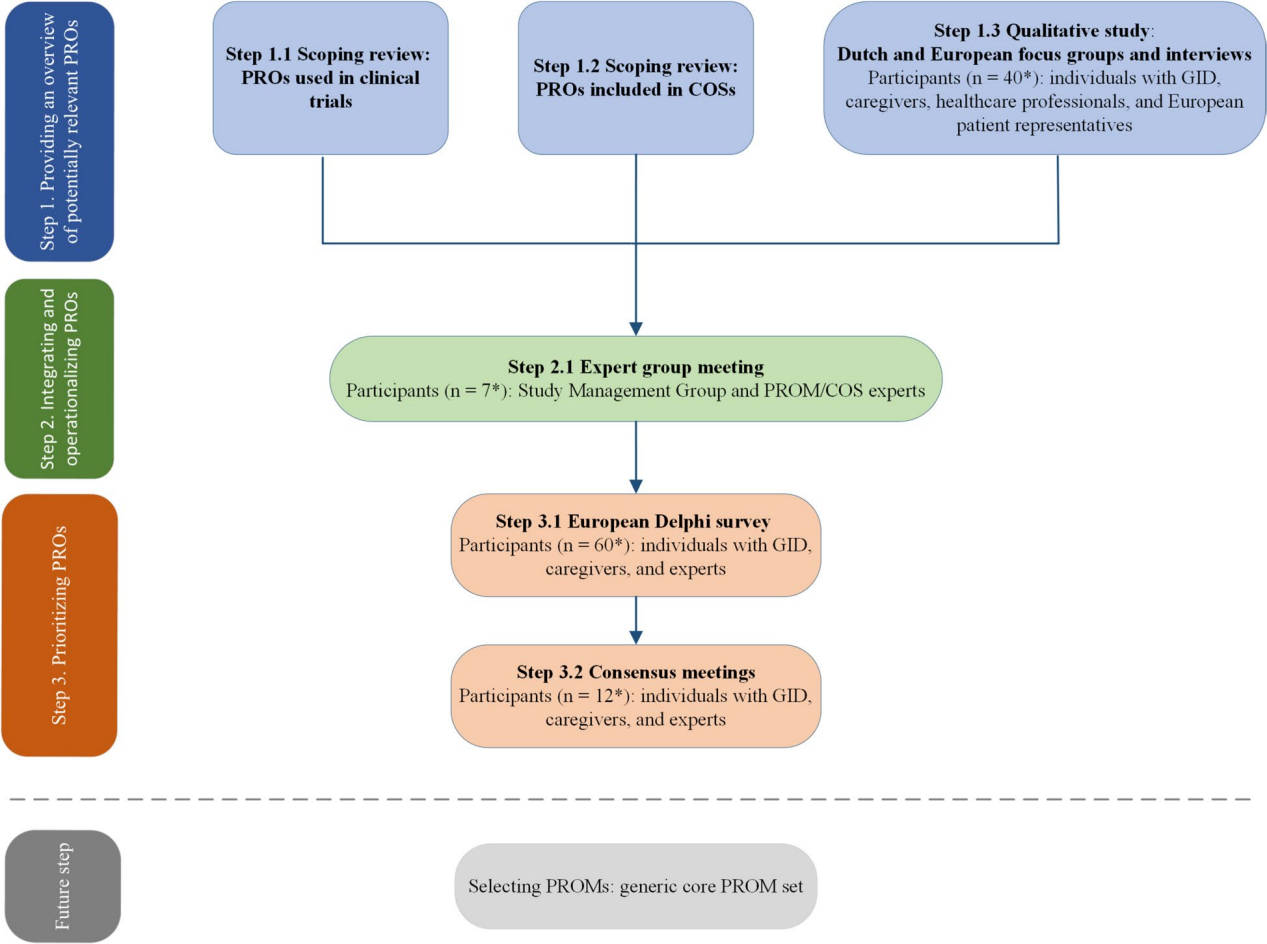
Steps for developing the generic core PRO set. PROs, patient reported outcomes; PROM(s), patient reported outcome measure(s); COS(s), core outcome set(s). *Target number of participants

Developing the generic core PRO set will consist of three distinct steps as described in Fig. 2.

### Step 1: Providing an overview of potentially relevant PROs

#### Step 1.1: Scoping review; PROs included in clinical trials

PROs used in clinical trials with GID will be identified. Data will be extracted from a previous published scoping review on outcomes and outcome measurement instruments used in clinical trials with individuals with genetic neurodevelopmental disorders and ID. The method and search strategy can be found in the original publication [34].

In our data extraction, only studies focusing on PROs and/or PROMs will be included. Studies including individuals with inborn errors of metabolism will be excluded, as these individuals lie outside the intended scope of the generic core PRO set. The subsequent data will be extracted: PRO(s), dimensionality PRO(s), PROM(s), type of PROM report (self-report or proxy-report), and type of PROM (generic, condition-specific, or individualized). PROs will be classified within the conceptual framework by the Study Management Group.

#### Step 1.2: Scoping review; PROs included in COSs

In the scoping review, PROs included in COSs for specific rare genetic neurodevelopmental disorders and/or ID will be identified. This review will be performed according to methodology published in advance in the Open Science Framework (OSF) [41].

MEDLINE (Ovid), PsycINFO, Embase, and the COMET database will be searched. A list of GID will be composed using the human phenotype ontology (HPO) database on https://hpo.jax.org/app/. All terms describing a genetic disorder assigned to the subontology ID will be included. Furthermore, a search strategy for ID of unknown cause will be used in combination with terms for a COS.

Any type of COS development studies for individuals with a rare genetic neurodevelopmental disorder or ID will be included. COS studies for application in research studies, for the use in care, and for the use of quality assessment will be included. COS studies covering all types of interventions will be included.

All titles and abstracts will be screened. Of the relevant articles, full texts will be reviewed and the same data will be extracted as in step 1.1. PROs will be classified within the conceptual framework by the Study Management Group. The psychometric properties of the PROMs will be checked for reliability and validity among individuals with GID.

#### Step 1.3: Qualitative study; Dutch and European focus groups and interviews

Focus groups and interviews will be conducted with affected individuals, their caregivers, healthcare professionals (e.g., medical doctors, psychologists, and nurses), and European patient representatives in order to qualitatively identify relevant PROs for individuals with GID. Affected individuals and caregivers will be recruited via their healthcare professional at the involved healthcare organizations (Amsterdam UMC and ‘s Heeren Loo). Healthcare professionals will be recruited through a call to participate in a focus group or interview at the Amsterdam UMC and ‘s Heeren Loo, and will be asked to disseminate the call to colleagues. European patient representatives will be recruited via ERN-ITHACA. Participants will be assessed for socio-demographic characteristics to ensure a representative sample.

Participants with GID will be selected using purposive sampling, aiming for diversity in age, genetic diagnoses, and level of ID [42]. To be eligible for inclusion in either a focus group or interview, individuals must be affected by GID, possess effective communication skills to express their thoughts and feelings to other participants or the interviewer, be over 12 years of age, and be proficient in Dutch (speaking, reading, and writing). Caregivers who provide care and support to an individual with a GID, as well as healthcare professionals with a minimum of five years’ experience working with individuals with a GID, will also be invited to participate. European patient representatives should have at least five years’ experience working for a patient organization dedicated to GID. The final number of participants recruited for the focus groups and interviews will be around 40, but the final number will depend on adequately sampling of the group until data sufficiency is reached.

Dutch focus groups and interviews will be performed at the Amsterdam UMC in the Netherlands via live and/or online meetings. European focus groups will be performed online. The duration of focus groups will be about 1.5 hours and interviews 30 minutes. The focus groups and interviews will consist of two parts:

- Part 1 – By using the ‘Complain and Cheer wall’ technique [43] (i.e., writing down negative aspects on the ‘Complain wall’ and positive aspects on the ‘Cheer wall’), the impact of a GID on daily life will be discussed.
- Part 2 – By using the ‘Brainstorming’ technique [43] (i.e., generating and discussing new ideas together), relevant topics regarding physical, mental and social functioning to discuss with the healthcare professional during a consultation will be discussed.

The PROs emerging from the focus groups and interviews will be classified within the conceptual framework by the Study Management Group.

### Step 2: Integrating and conceptualizing PROs

#### Step 2.1 Expert group meeting

The PROs established and classified in step 1.1 to step 1.3 will be integrated into a pilot generic core PRO set. PROs will be eliminated where appropriate (e.g., duplicate or no PRO, such as behavior) and multidimensional PROs will be separated into unidimensional PROs whenever possible. For example, the multidimensional PRO ‘adaptive functioning’ will be separated into unidimensional PROs: ‘communication’, ‘daily living skills’, and ‘social functioning’. The PROs will be conceptualized to ensure clear PRO definition. Shared experiences identified by the focus groups and interviews, as well as those found in the literature and guidelines, will be used to conceptualize the PROs. This approach aims to ensure the applicability of the PRO conceptualizations to all GID groups. As experiences are meant to be generic, we expect them to remain manageable and describable within the conceptualization of a generic PRO. Eventually, a reduced and clearly defined list of PROs will remain, classified into the conceptual framework: the pilot generic core PRO set. Step 2.1 will be performed by the Study Management Group and two PROM/COS experts.

### Step 3: Prioritizing the relevant PROs

#### Step 3.1: European Delphi survey

A European Delphi survey will be performed to reach consensus on which PROs should be included in the final generic core PRO set. A Delphi survey is a technique used to gain consensus among relevant experts or stakeholders. The Delphi method typically involves multiple rounds in which participants remain anonymous in the surveys [44].

Participant groups will align with those in the focus groups and interviews. Healthcare professionals and European patient representatives will be merged into one stakeholder group: experts. Participants of the Dutch and European focus groups and interviews will be contacted again if they have given their consent. Additional individuals with GID, caregivers, and experts will be contacted through Dutch patient organizations dedicated to a specific GID and ERN-ITHACA. To ensure a representative sample, the socio-demographic characteristics of the participants will be checked.

There is currently no established guideline for determining the optimal sample size for a Delphi survey [45, 46]. The Study Management Group will aim at including 60 participants in the Delphi survey in order to represent the GID population as best as possible, ensuring the representation of 20 individuals with GID, 20 caregivers, and 20 experts. The pilot generic core PRO set will be presented in the Delphi survey. The survey will be kept as concise as possible. Additionally, participants, especially affected individuals, will have the option to complete the survey at multiple times to prevent overburdening. The Delphi survey will first be pilot-tested with one member of each participant group to ensure that the survey is clearly understandable.

#### Round one

In the first survey round, PROs will be prioritized. The pilot generic core PRO set will be presented to participants, including a list of excluded PROs or other outcomes along with the reason for their exclusion. Participants will be asked to determine why a PRO is important enough to be included in the generic core PRO set, or why a PRO should be excluded: response options will be ‘Yes’, ‘Unsure/I do not know’, and ‘No’. They may also provide a rationale for their decision, respond to the proposed conceptualization of the PROs to ensure that no shared experiences are overlooked, and will be asked for additional PROs.

The ‘70/15%’ consensus definition (i.e., including an outcome in the COS when at least 70% of participants score an outcome as ‘very important’ and less than 15% as ‘not important’), which is described by Williamson et al. (2012) [47], has previously been used by other COS developers [48–50]. In addition, the COnsensus-based Standards for the selection of health Measurement INstruments (COSMIN) initiative usually use a consensus threshold of 67% in Delphi studies [51, 52]. However, given the considerable diversity within and between GID subgroups, it is reasonable to anticipate a wide range of relevant PROs. For this reason, the consensus threshold will not be excessively high: the consensus threshold will be set at 60% or more of all stakeholder groups responding ‘Yes’ for a PRO to include the PRO in the final generic core PRO set. If 60% or more of all stakeholder groups respond ‘No’ for a PRO, it will not be included in the final generic core PRO set. The responses of round one will be analyzed for each participant group. The PROs that have reached consensus for inclusion or exclusion from the final generic core PRO set will be shown again in the second round to allow participants to reassess them and ensure their inclusion or exclusion. If at least two participants suggest the same modification to the conceptualization of a PRO or suggest the same additional PRO in the open-ended question, it will be included in the second round.

#### Round two

In the second survey round, PROs will be reprioritized with the aim to reach consensus on which PROs should be included in the final generic core PRO set. The pilot generic core PRO set, along with feedback from the first round, will be presented to participants. The results for each PRO will be aggregated for each participant group, and summary statistics and reason for inclusion or exclusion will be presented. Response options will be the same as in the first round. The responses of round two will be analyzed again for each participant group. Responses will be carefully examined to ensure no strong objections are raised against the prevailing group response.

#### Step 3.2: Two consensus meetings

Two separate consensus meetings will be organized to reach consensus on the undecided PROs: one online meeting with three Dutch individuals with GID and three caregivers, and one online meeting with six experts. At the end of Delphi round two, participants will be invited to join a consensus meeting. Those who wish to participate will be checked on socio-demographic characteristics to ensure diverse representation in the consensus meetings. PROs that have not reached consensus during the Delphi will be subjected to voting in these two meetings. A PRO receiving at least 60% positive votes during both meetings will be included in the final generic core PRO set.

## Discussion

To our knowledge, the current study is the first to develop a generic core PRO set for children and adults with GID. An innovative and thorough methodology is used, involving the identification, classification, and conceptualization of PROs within a comprehensive conceptual framework. This methodology may also be used for the development of generic core PRO sets for other (rare) health conditions. With this study, we aim to reach an important milestone in addressing the needs of individuals with GID. The most relevant PROs for the whole GID population will be identified. Subsequently, a generic core PRO set will be developed that should be consistently measured in both care and research, all from the perspective of affected individuals or their caregivers.

For the development of the generic core PRO set, an all-encompassing conceptual framework will be used to classify the PROs for GID. An essential and novel step is integrating and conceptualizing PROs into this conceptual framework in step 2.1 (the pilot generic core PRO set). PROs often lack precise definition or exhibit multidimensionality, a common occurrence when they are presented in Delphi surveys. This makes it challenging for Delphi participants to grasp the exact meaning of the PROs. By integrating and conceptualizing PROs, we aim to include a reduced, well-defined, but still all-encompassing list of unidimensional PROs (whenever possible) in the Delphi survey. This approach not only ensures that participants understand and recognize the meaning of the PROs, enabling them to provide thoughtful responses for each PRO, it also facilitates the process of eventually selecting suitable PROMs, as it becomes very clear ‘what’ needs to be measured. While this is a crucial step, we are aware it will be challenging to incorporate and conceptualize all potentially relevant PROs for the whole GID population. Nevertheless, diligently performing the steps outlined in this protocol should result in a classified and well-defined generic core PRO set relevant to the whole GID population. Moreover, it will also become clear which outcomes do not qualify as a PRO, and should therefore be measured with alternative instruments. The next step involves the selection of suitable PROMs with optimal psychometric properties which sufficiently measure the generic core PRO set: the generic core PROM set. As GID is a heterogeneous population, condition-specific PROs will be necessary for specific GID subgroups. Additionally, there may also be a need for specific PROs for other clinically significant subgroups (e.g., age, ID level) that are not sufficiently covered by the generic core PRO set. Therefore, it is crucial to explore these subgroups to identify potential condition-specific or other subgroup-specific PROs. If necessary, specific questions or PROMs for specific genetic conditions or other subgroups will be added. Subsequently, the generic core PROM set will be validated within the GID population (e.g., by measuring reliability, internal consistency, and responsiveness of the PROMs), and implemented in care and research. The validation and implementation of the core PROM set will be done in collaboration with experienced methodologists and implementation scientists [53–55]. Consideration will be given to known PROM implementation barriers [55–57].

### Necessity of a core PRO(M) set

The development of the generic core PRO(M) set forms the first step in standardizing personalized measurement in care and research for children and adults with GID [58]. This novel and state-of-the-art generic core PRO(M) set will enable consistent and standardized measurement of relevant PROs across different GID groups in both care and research. This reduces the great diversity of PROs measured, simplifying the aggregation and analysis of PRO data. Moreover, this approach ensures an ongoing integration of patient perspective in care and research, leading to a better understanding of the overall impact and the variations in impact on daily functioning among different GIDs, or other subgroups of interest such as those affected by shared comorbidity such as autism spectrum disorders. The necessity to integrate PRO data, especially in research areas such as drug development and disease-modifying therapies [59], resounds not only from patients themselves, but also from healthcare professionals and policymakers including the European Medicines Agency (EMA) and the Food and Drug Administration (FDA) [60–63]. Furthermore, utilizing the generic core PRO(M) set will also tackle the ongoing challenge in finding suitable PROMs for individuals with GID. PROMs can be cognitively demanding, as many affected individuals encounter difficulties understanding and responding to questions [64], and PROMs frequently consist of lengthy and difficult questions. In addition, PROMs are often not tested and validated within the GID population or only in a specific population group [30]. This raises the questions about whether the current PROMs used for GID accurately measure real treatment effects and progress of affected individuals [65]. At last, utilizing the generic core PRO(M) in care and research settings may offer many benefits for the individual with GID and the GID population as a whole (Fig. 1).

## Conclusions

This protocol outlines the steps for developing a generic core PRO set for children and adults with GID. The subsequent step focuses on the careful selection of PROMs to effectively measure these PROs, forming the generic core PROM set. This generic core PROM set needs to be validated in the GID population. Future utilization of such a generic core PROM set in both care and research, will facilitate aggregation and comparison of PRO data and guarantee the integration of the patient perspective.

## Supplementary Information

Supplementary Material 1.

## Data Availability

Not applicable.
