# Supplementary material for: PROs for RARE: protocol for development of a core patient reported outcome set for individuals with genetic intellectual disability
Source: Orphanet J Rare Dis. 2024 Sep 27;19:354. doi: 10.1186/s13023-024-03264-0 (PMC11428331; doi:10.1186/s13023-024-03264-0)
Supplement: Supplementary file 1 — Supplementary Material 1. [file 13023_2024_3264_MOESM1_ESM.pdf]

Additional file 1. Glossary of terms

Abbreviation	Full text	Definition	Examples
PRO	Patient reported outcome	Aspects of a patient's health status that are directly reported by patients themselves or a proxy, without interference of a clinician or someone else	Anxiety, anger
Unidimensional PRO		A PRO including a single PRO construct	Pain, fatigue
Multidimensional PRO		A PRO including multiple PRO constructs	Quality of life, adaptive functioning
PROM	Patient reported outcome measure	A standardized questionnaire used to measure a PRO	PROMIS pediatric global health
Generic PROM		Measuring health concepts that are relevant to a broad range of conditions or the general population	Pediatric Quality of Life Inventory; 36-Item Short Form Survey
Condition-specific PROM		Measuring health concepts relevant to a specific condition	TSC-PROM; PedsQL Neurofibromatis type 1 Module
Individualized PROM		Measuring health concepts relevant to a specific individual	Patient-Specific Complaint Questionnaire
COS	Core outcome set	An agreed standardized set of outcomes which should be measured and reported in all	COS for Dravet syndrome

		clinical trials for a specific condition	
Core PRO set	Core patient reported outcome set	An agreed standardized set of relevant PROs which should be measured and reported in care and research	Core PRO set for GID (current project)
